# Local tuning of radiomics-based model for predicting pathological response to neoadjuvant chemoradiotherapy in locally advanced rectal cancer

**DOI:** 10.1186/s12880-022-00773-x

**Published:** 2022-03-14

**Authors:** Bin Tang, Jacopo Lenkowicz, Qian Peng, Luca Boldrini, Qing Hou, Nicola Dinapoli, Vincenzo Valentini, Peng Diao, Gang Yin, Lucia Clara Orlandini

**Affiliations:** 1 Key Laboratory of Radiation Physics and Technology of the Ministry of Education, Institute of Nuclear Science and Technology, Sichuan University, Chengdu, China; 2 Department of Radiation Oncology, Radiation Oncology Key Laboratory of Sichuan Province, Sichuan Cancer Hospital and Institute, Chengdu, China; 3 Dipartimento Scienze Radiologiche, Fondazione Policlinico Universitario “A. Gemelli” IRCCS, Rome, Italy

**Keywords:** Radiomics, Rectum, Predictive models, Pathological complete response, LASSO

## Abstract

**Purpose:**

This study aims to further enhance a validated radiomics-based model for predicting pathologic complete response (pCR) after chemo‑radiotherapy in locally advanced rectal cancer (LARC) for use in clinical practice.

**Methods:**

A generalized linear model (GLM) to predict pCR in LARC patients previously trained in Europe and validated with an external inter-continental cohort (59 patients), was first examined with further 88 intercontinental patient datasets to assess its reproducibility; then new radiomics and clinical features, and validation methods were investigated to build a new model for enhancing the pCR prediction for patients admitted to our department. The patients were divided into training group (75%) and validation group (25%) according to their demographic. The least absolute shrinkage and selection operator (LASSO) logistic regression was used to reduce the dimensionality of the extracted features of the training group and select the optimal ones; the performance of the reference GLM and enhanced models was compared through the area under curve (AUC) of the receiver operating characteristics.

**Results:**

The value of AUC of the reference model was 0.831 (95% CI, 0.701–0.961), and 0.828 (95% CI, 0.700–0.956) in the original and new validation cohorts, respectively, showing a reproducibility in the applicability of the GLM model. Eight features were found to be significant with LASSO and used to establish an enhanced model. The AUC of the enhanced model of 0.926 (95% CI, 0.859–0.993) for training, and 0.926 (95% CI, 0.767–1.00) for the validation group shows better performance than the reference model.

**Conclusions:**

The GLM model shows good reproducibility in predicting pCR in LARC; the enhanced model has the potential to improve prediction accuracy and may be a candidate in clinical practice.

## Background

For locally advanced rectal cancer (LARC), the standard-of-care treatment is preoperative neoadjuvant chemoradiotherapy (nCRT) followed by total mesorectal excision (TME). While TME remains the gold standard, it is associated with significant morbidity and long-term effects on anorectal, urinary, and sexual function [1, 2]. Despite the consensus on this treatment schedule, the response of these tumors is heterogeneous, with approximately 20% of patients showing a pathologic complete response (pCR) [3, 4]; which might be indicative of a prognostically favorable biological tumor profile with less propensity for local or distant recurrence and improved survival [5]. Therefore, in those who achieve a pCR, some researchers have questioned the use of TME surgery and investigated the appropriateness of proceeding with a partial resection, or even omitting surgery while undertaking intensive follow-up [6, 7]. It is critical to be able to early identify those patients who will have a complete clinical response to nCRT, which has remained a topic of research for many years. Tumor-related factors including clinical, pathological, radiological and molecular markers were studied for the prediction of pCR [8–10], however, no robust markers have been identified so far.

Recently, radiomics has emerged as a viable and powerful tool for diagnostic and prognostic purpose [11]. The term radiomics refers to the extraction and analysis of features from medical images acquired by proton emission tomography, computed tomography, magnetic resonance (MR), etc., to build descriptive, diagnostic, or predictive models. These medical images effectively carry an immense source of potential data for decoding tumor phenotypes [12]. The strength of radiomics lies in the wide use and non-invasiveness of medical imaging in clinical routine. The translation of radiomics analysis into standard cancer care to support treatment decision-making involves the development of prediction models integrating clinical information that can assess the risk of specific tumor outcomes [11].

In the case of radiomic-based prediction of pCR in LARC, many studies have developed predictive models based on clinical medical images. Bundschuh et al. [13] extracted textural features from pre-treatment 18F-FDG PET/CT and the coefficients of variation showed the capability to assess pCR in 27 patients. Lovinfosse et al. [14] found textural features of 18F-FDG PET/CT especially coarseness had better predictive power than intensity- and volume-based parameters for pCR prediction in a cohort of 86 LARC patients. While non-contrast CT is not diagnostically used for LARC, Hamerla et al. [15] concluded that no added value of a radiomics model based on non-contrast CT for prediction of pCR. MRI with superior soft tissue contrast is the current standard in the assessment and staging of rectal cancer, thus many radiomics based prediction is conducted on MRI, and the multiparametric nature of MRI further attracted growing research interest. Zhou et al. [16] studied multiparametric MRI-based model based on including T1-weighted (T1w), T2-weighted (T2w), diffusion-weighted imaging (DWI) and contrast-enhanced T1-weighted to predict pCR. De Cecco et al. [17] extracted the texture parameters of pre- and after-treatment T2w MRI acquired on a 3 T scanner, and found that the quantitative change of texture parameters have the potential to act as imaging biomarkers of pCR. Boldrini et al. [18] also suggested the change of the features of daily images throughout the treatment in 0.35 T MRI-guided radiotherapy can discriminate between pCR and non-pCR patients.

Our work originated in a previously developed magnetic resonance, vendor-independent radiomics based model [19]. This model, as a reference model, was developed using pre-treatment T2w MRI acquired on a 1.5 T scanner in Europe. Here we aim to evaluate its reproducibility and generalization with pre-treatment 3 T T2w MRI of LARC patients admitted to our center. Moreover, the reference model only adopted geometrical and intensity-histogram features, while a lot of other radiomics features also show the potential to act as biomarkers in some studies [20–24]. We investigated the significance of a wider range of radiomics features to the pCR status and build a new radiomics model to further enhance its prediction of the response to nCRT for LARC patients admitted to our institute.

## Methods

### Reference model

A generalized linear model (GLM) [19], which was built to predict pCR in LARC patients using a single-center training set of 162 patients and 2 external validation sets of 34 and 25 patients respectively provided by other European centers, was used as the reference model in this study. The model is magnetic resonance (MR) vendor-independent and based on four predictors: clinical T and N staging and two radiomics features (Skewness and Entropy) extracted from staging 1.5 T MRI. The pCR achievement was considered as the binary outcome. Predictive performance of the model, evaluated by the Area under Curve (AUC) of the Receiver Operating Characteristic (ROC) showed an AUC of 0.73 (95% CI 0.65–0.82) in the training cohort and 0.75 (95% CI 0.61–0.88) in the testing cohort. Successively, the model was validated with an inter-continental cohort of 59 patients from our Institute showing an AUC of 0.831 (95% CI, 0.701‐0.961) [25].

### Patients

A total of 88 patients pathologically confirmed locally advanced rectal adenocarcinoma, clinical stage T3-4N0 or T1-4N1-2 and treated in Sichuan Cancer Hospital & Institute between March 2017 and December 2020, were enrolled in this retrospective study. This study was approved by the Ethics Committee of Sichuan Cancer Hospital (approval number SCCHEC-02-2020-008). The need for informed written patient consent was waived due to the retrospective nature of this study; nevertheless the patients gave oral consent to the use of their anonymized data for research purposes.

Patients with distant metastases, prior chemotherapy, or radiotherapy for rectal cancer, previous or concurrent malignancies, and known allergies to intravenous contrast agents or other contraindications for MR imaging (MRI) acquisition were excluded. All patients received MR examinations one week before preoperative chemoradiation. Two treatment protocols were as follows: first, a one-week short course of external radiation therapy (EBRT, 25 Gy in 5 doses of 5 Gy each); second, a long course of 5–6 weeks of EBRT (50.4 Gy in 28 fractions of 1.8 Gy each) concurrently with chemotherapy (Capecitabine 825 mg/m^2^ die). After one of the two treatment protocols was administered, 3–4 cycles of Capoex chemotherapy (oxaliplatin 130 mg/m^2^ d1 + capecitabine 1000 mg/m2, d1-14) was performed. There was a 6–8 week break followed by TME and postoperative 4–5 cycles of Capoex chemotherapy. TME was performed by either anterior resection or abdominoperineal resection. The pathologic staging served as the reference standard and was determined according to the TNM classification system recommended by the American Joint Committee on Cancer (AJCC), 7th ed., 2012 [26]. The resection specimens were evaluated by an experienced pathologist blinded to the MRI data. Response to nCRT was determined by histopathological examination of surgically resected specimens: tumour responses were classified using tumor regression grade (TRG) according to Mandard et al. [27] as pCR (TRG = 1), or non-responder (TRG > 1).

### Magnetic resonance imaging

All patients were scanned in our institute with a 3.0 Tesla MR (Siemens Skyra, Siemens Medical Systems) scanner, using a phased-array body coil one week before the start of chemoradiation with fixed image protocols. No special bowel preparation was performed. The MR machine underwent quality assurance check monthly by the medical physics department with particular attention to the image’s quality controls. The scanning protocol followed by the patient and used for this study consists of an axial T2-weighted fast spin-echo sequence, with 2840 ms repetition time and 131 ms echo time, traversal image resolution 0.625 × 0.625 mm, slice spacing 3.85 mm and slice thickness 3.5 mm.

### Features extraction

All MR images were reviewed in MIMMaestro workstation (MIM Software Inc, Cleveland, OH) by a 10-year experienced rectal MRI radiologist who delineated the gross tumor volume (GTV) following the guidelines defined in ICRU n.83 [28]. The segmentation process was performed manually and the radiomics analysis was focused on the entire volume. All DICOM files containing the MR images and the corresponding radiotherapy (RT) Structure files were imported in Moddicom, an open-source R (R Core Team, Vienna, Austria) statistical software package [29]. Images were pre-processed with the Laplacian of Gaussian (LoG) convolution kernel filter to decrease the high-frequency MRI signal noise and reduce the impact of large variations of signal. The size of the standard deviation (σ) in the LoG filter was scanned from 0.1 to 1.0 with a step-size of 0.05. To search for potential GTV features related to outcome prediction, five groups of features were extracted from the GTV on each pre-processed image, including statistical, morphological, grey-level co-occurrence matrix (GLCM), grey-level run length matrix (GLRLM), grey-level size zone matrix (GLSZM). In addition, three potential clinical features, i.e., clinical T-stage (cT), clinical N-stage (cN) and age, were also acquired for later analysis.

### Feature selection and LASSO regression model

A two-step process was applied to feature selection. First, for all features extracted from pre-processed T2w images with variant LoG filters and three clinical features, the Mann–Whitney U-test was used to find potential features significant for pCR. Second, to further reduce the number of final feature predictors and avoid multicollinearity between them, the binary logistic regression model LASSO (least absolute shrinkage and selection operator) was used to search an optimal subset of features from those screened out by Mann–Whitney test. By increasing the lambda parameters incorporated in the LASSO model, more non-zero coefficients of the features were set to 0, so fewer features would be chosen. Meanwhile, the logistic regression model was established between chosen features and the pCR. The variation of the subset of features with their corresponding coefficients in the model changes the AUC of the ROC. With fivefold cross-validation, the best lambda counterpart with the highest AUC was selected. The 95% confidence interval of the AUC of each ROC was computed using bootstrap method with 1000 resamplings. Also the Rad-score was constructed with the final subset of features with the following equation:

$${\text{Rad - score}} = \mathop \sum \limits_{i}^{n} C_{i} X_{i} + b$$

where *n* represents the total number of features, *X*_*i*_ is the *i*th feature, *C*_*i*_ is the coefficient of *X*_*i*_ and *b* is the intercept.

## Results

### Patient characteristics

In this study, we enrolled 88 LARC patients who underwent standard CRT, including 12 (13.6%) responders and 76 (86.4%) non-responders to validate and enhance the performance of the reference model. Table 1 summarized patient tumors characteristics and outcomes. Statistical results investigating significant differences were reported in the last column: chi-square test was performed for categorical variables, Wilcoxon Mann Whitney for continuous ones. There were no significant differences in clinical variables between the original cohort used to validate the reference model and the new cohort used to set up the enhanced model. While the regimens and TRG show significant differences between two cohorts (*p* < 0.05).

**Table 1:** Patient and tumour characteristics, clinical data, and response outcome

	Original cohort	New cohort	*p* value
Number	59	88	
Age			0.387
Years, median (range)	56.0 (34.0–74.0)	55.5 (29.0–73.0)	
Sex—no. (%)			0.565
Male	47 (79.7)	63 (71.6)	
Female	12 (20.3)	25 (28.4)	
Tumor stage—no. (%)			
cT stage			0.239
T2	6(10.2)	2 (2.3)	
T3	34 (57.6)	61 (69.3)	
T4	19 (32.2)	25 (28.4)	
cN stage			0.365
N0	25 (42.4)	29(32.9)	
N1	24 (40.7)	21(23.9)	
N2	10(16.9)	38 (43.2)	
Interval between MRI and start CRT			< 0.05
Days, median (range)	14 (4–50)	13 (4–35)	
Interval between end CRT and surgery			< 0.05
RT Short Course: days, median (range)	10 (8–15)	9(5–15)	
RT Long Course^a^: days, median (range)	59(30–82)	67(30–108)	
RT Course			< 0.05
Short (5fr x 5 Gy)—no. (%)	19 (32.2)	10 (11.4)	
Long (28fr × 1.8 Gy)—no. (%)	39 (67.8)	78 (88.6)	
eMR scanner Strength			< 0.05
1.5 T no (%)	32 (54.2)		
3.0 T no (%)	27 (45.8)	88 (100.0)	
TRG			< 0.05
1—no. (%)	10 (16.9)	12 (13.6)	
2–5—no. (%)	49 (83.1)	76 (86.4)	

^a^In the long radiotherapy course, two more chemotherapy cycles were scheduled at the end of the radiotherapy before surgery

### Feature selection and rad-score construction

**Fig. 1:**
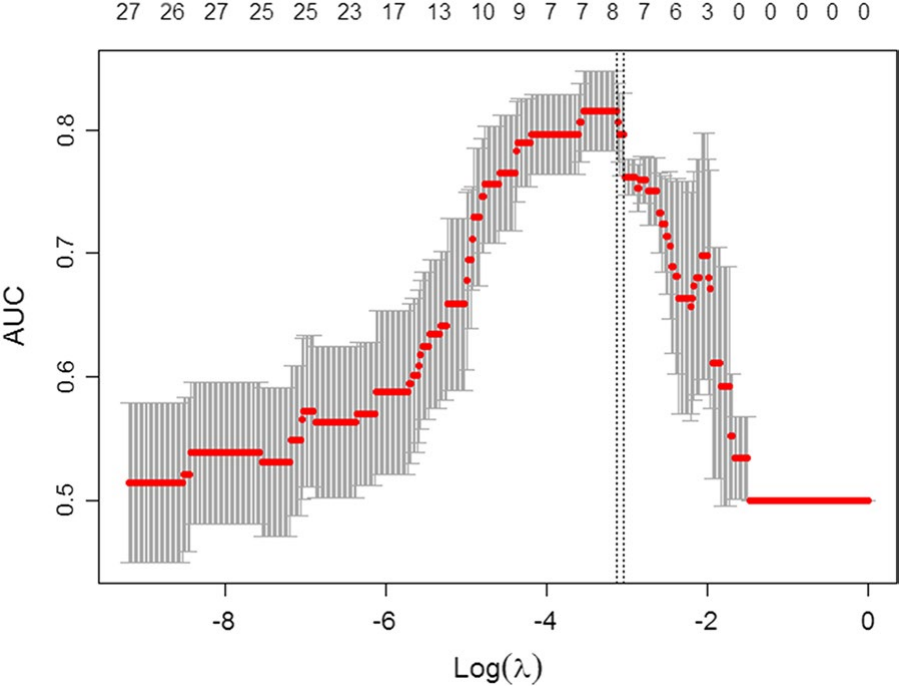
Selection of the optimal lambda value for the enhanced LASSO model

A total of 1643 features were obtained from the LoG filtered T2-weighted MR images. Sixty features were found significant for pCR in the Mann–Whitney U test. In the LASSO model, λ was chosen by fivefold cross-validation, and log(λ) of − 3.13 was the optimal subset for one clinical feature (age) and seven radiomics features i.e. surface to volume ratio, sum variance, cluster tendency, entropy, high grey level run emphasis, sum entropy, high grey level run emphasis 1, and mean intensity with LoG filters of variant sigma, as listed in Table 2. Figure 1 highlights how the number of variables contained in the model varies with the lambda parameter.

**Table 2:** The coefficient, and sigma of LoG filter for the eight features adopted in the enhanced prediction model

Feature name	Sigma of LoG filter	Coefficient
Sum entropy	0.65	1.45E0
Surface to volume ratio	0.7	5.67E−01
Entropy	0.5	− 1.46E−01
Age	–	− 7.7E−03
High grey level run emphasis	0.6	1.27E−03
Sum variance	0.65	1.26E−03
Mean intensity	0.65	− 4.57E−04
High grey level run emphasis 1	0.6	3.16E−07
Cluster tendency	0.65	2.37E−17

**Fig. 2:**
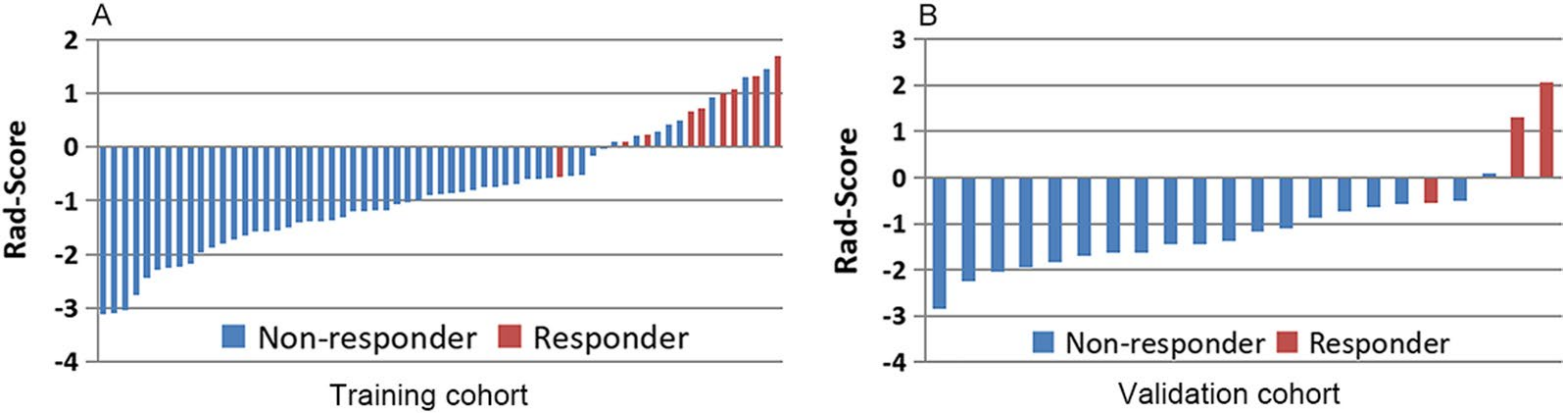
The radiomics score of the enhanced model for patients in (**A**) the training cohort and (**B**) the validation cohort

The Rad-score was calculated for each patient based on the linear combination of eight features with their respective coefficients. Waterfall plots showed the Rad-score for individuals in the training cohort (Fig. 2A) and validation cohort (Fig. 2B). There was a significant difference in rad score between pCR and non-pCR group in both the training (*p* < 0.001) and the validation cohort (*p* < 0.003).

### Performance comparison

**Fig. 3:**
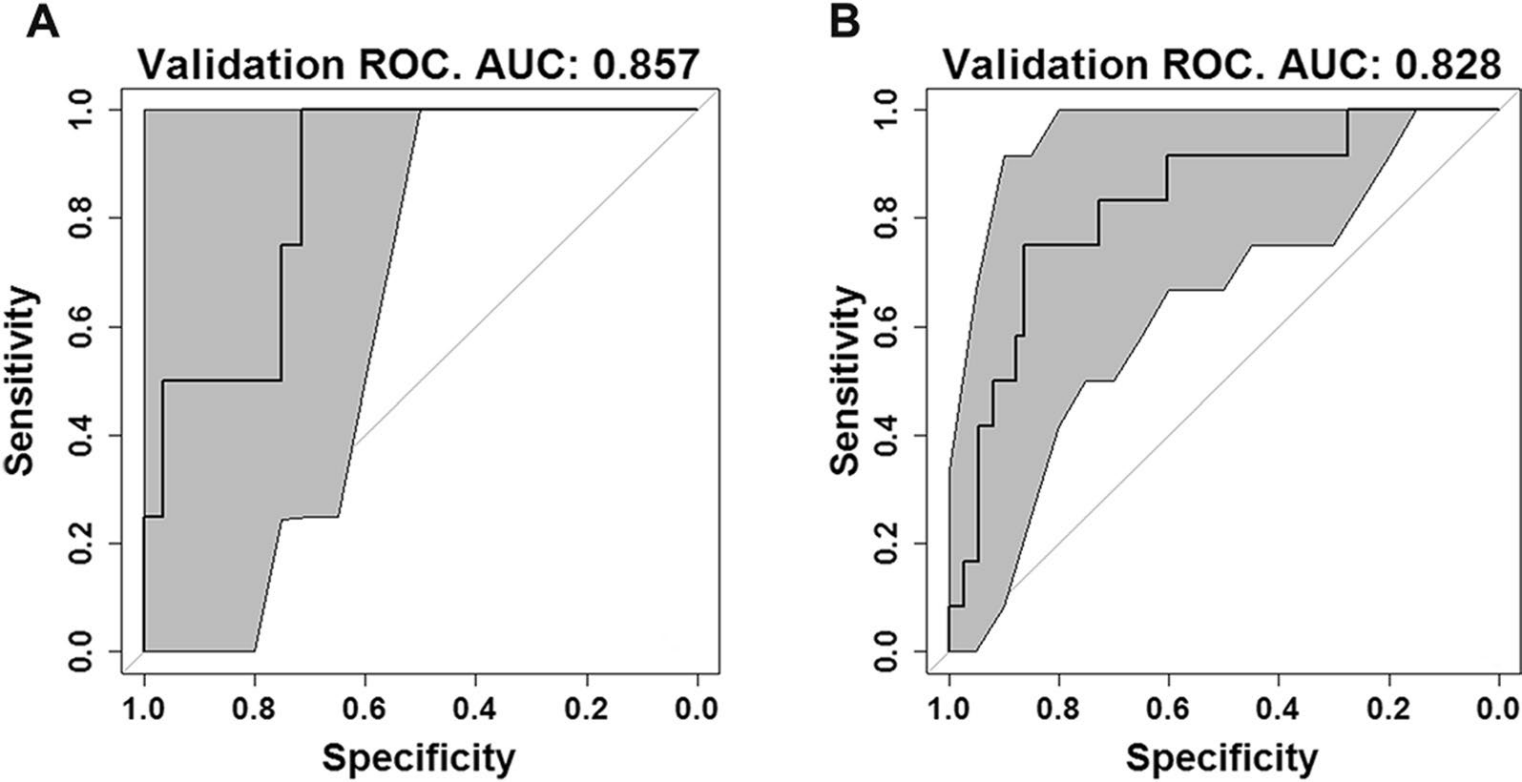
ROC curve of the reference model (GLM) for the original (**A**) and new (**B**) validation cohort of patients

**Fig. 4:**
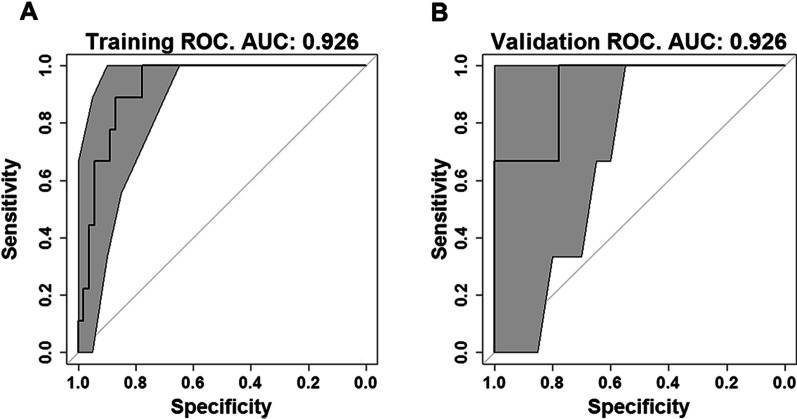
ROC curves of the enhanced model (LASSO) for the training (**A**) and validation (**B**) group of the new cohort of patients

The AUC value of the ROC curves of the reference model for the original cohort and new cohort of patients are shown in Fig. 3; the AUC of 0.831 (95% CI, 0.701–0.961), and 0.828 (95% CI, 0.700–0.956) in the original and new validation cohorts, respectively, showed reproducibility in the applicability of the original model; whereas the AUC value of the ROC curves of the enhanced model portrayed in Fig. 4 is 0.926 (95% CI, 0.859–0.993) for the training and 0.926 (95% CI, 0.767–1.00) for the validation group.

## Discussion

MRI after nCRT usually has the problem of over-staging for patients with a pCR because of difficulties in differentiating desmoplastic reaction caused by fibrosis alone or fibrosis that contains tumor cell [30, 31], thus pathological evaluation of the surgical specimen is the only reliable surrogate marker that correlates with long-term oncological outcomes. However, such data are only available after completion of all preoperative treatments and surgery and cannot be used to guide the therapeutic approach. Therefore, the development of non-invasive biomarkers with the capacity to provide early prediction is essential. Such biomarkers would help to identify patients who are less likely to benefit from current therapies as they are more likely to have a pCR. Radiomics, as an emerging non-invasive predictive biomarker, has been proven valid in many scientific fields, including the prediction of pCR for LARC patients.

The reproducibility is a big concern in the field of radiomic study since the features extracted from MR images could be impacted by patients, sequences, acquisition parameters, or simply time [32]. In this study, we first validated the previous GLM prediction model [19] with an intercontinental cohort enrolled in our hospital. Although the cohort in this study used 3 T T2-weighted pre-nCRT MRI images which is different from the modality used for training in GLM model, this fact might potentially interfere with the reproducibility of model performance [33]. Table 1 reported there were indeed significant differences of regimens and TRG between two cohorts. Nevertheless, a good result was still achieved, which demonstrated the reproducibility and generalization of GLM model.

As for radiomic features which are significant to pCR prediction, the findings vary from study to study. This is partly because of the heterogeneity of MRI modality utilized in those studies. For example, De et al. [17] found the change of Kurtosis, MPP and Skewness of 3 T T2w MRI throughout treatment is significant for the prediction of pCR; Li et al. [21] found the difference of Skewness, Run length Non-uniformity, Local Entropy Max, Local Range Min Coarseness, Maximum 3D diameter, Surface Area Density between pre-nRT and post-nRT 1.5 T T1w/T2w MRI is significant; Zhou et al. [16] used Skewness, Mean, Median, entropy, dissimilarity and correlation, etc. of 1.5 T DWI, T1w/T2w and CE-T1w MRI for model building. While the reference paper (GLM model) for this study only explored the significance of first-order features from T2w MRI images, Skewness and Entropy were found significant and used for prediction.

The rationale for optimizing a model already validated in a cohort of patients from our hospital lies in the fact that the cohort of patients is representative of the patient population coming from our hospital. Hereby we expand the search range of features from first-order to morphological, GLCM, GLRLM, GLSZM of a cohort of patients admitted in our institution having MRI imaging with uniform characteristics and protocols, as well as the type of well-defined treatment course. Also, we add Age at the beginning of feature selection since some studies [16, 19, 34] show that the incorporation of clinical characteristics into the predictive model can improve model performance. Considering finding the most significant ones from a large number of initially extracted features, we adopted the LASSO model in this study. Compared to the previously developed GLM model, L1 regularization was added in the cost function of LASSO, which can reduce the dimensionality of radiomic features and avoid multilinearity. Finally, seven radiomics features and one clinical factor were selected after the regression coefficients of other features were penalized to zero. Comparing those predictors (features) in previous GLM and current LASSO, only Entropy of GTV is shared by both predictive models while other predictors are different. The finding of AUC of 0.926 indicates that LASSO indeed has an enhanced predictive performance compared to GLM.

The present study has some limitations. First, the sample size is still limited compared with the relatively large number of predictors. Nowadays there are many standardized features. With the pre-processing Log filter, the number of extracted features could increase to the level of thousand. Hopefully, we could include more patients in the future in addition to the cohort of 88 patients enrolled in this study. Second, the radiomic-based model developed here only utilized MRI T2w images. Although the use of one single MRI sequence could simplify the process of applying prediction tool in clinical routine, however, some other MRI sequences, i.e. diffusion-weighted image (DWI), show better delineation reproducibility and detection of tumor than T2w images [35, 36]. The inclusion of DWI or other MRI modalities in a radiomic study has the potential to further improve prediction performance.

## Conclusions

Previously GLM model show good reproducibility in predicting pCR to nCRT in LARC; The enhanced LASSO model developed in this study has the potential to improve prediction accuracy.

## Data Availability

The datasets generated during and analyzed during the present study are not publicly available due to participant privacy, but are available from the corresponding author on reasonable request.

## References

[1] Votava J, Kachlik D, Hoch J. Total mesorectal excision—40 years of standard of rectal cancer surgery Acta Chir Belg 2020, 120(4):286–290. 10.1080/00015458.2020.1745529 32200705

[2] Hajibandeh S, Hajibandeh S, Eltair M, George A T, Peravali R. Meta-analysis of transanal total mesorectal excision versus laparoscopic total mesorectal excision in management of rectal cancer. Int J Colorectal Dis 2020(12). 10.1007/s00384-020-03545-7 32124047

[3] Janjan NA, Khoo VS, Abbruzzese J, Pazdur R, Dubrow R, Cleary KR (1999). Tumor downstaging and sphincter preservation with preoperative chemoradiation in locally advanced rectal cancer: the M.D. Anderson Cancer Center experience. Int J Radiat Oncol Biol Phys.

[4] Pucciarelli S, Toppan P, Friso ML, Russo V, Pasetto L, Urso E (2004). Complete pathologic response following preoperative chemoradiation therapy for middle to lower rectal cancer is not a prognostic factor for a better outcome. Dis Colon Rectum.

[5] Maas M, Nelemans PJ, Valentini V, Das P, Rödel C, Kuo L-J (2010). Long-term outcome in patients with a pathological complete response after chemoradiation for rectal cancer: a pooled analysis of individual patient data. Lancet Oncol.

[6] Issa N, Murninkas A, Powsner E, Dreznick Z (2012). Long-term outcome of local excision after complete pathological response to neoadjuvant chemoradiation therapy for rectal cancer. World J Surg.

[7] Sanghera P, Wong DWY, Mcconkey CC, Geh JI, Hartley A (2008). Chemoradiotherapy for rectal cancer: an updated analysis of factors affecting pathological response. Clin Oncol.

[8] Barbaro B, Vitale R, Leccisotti L, Vecchio FM, Santoro L, Valentini V (2010). Restaging locally advanced rectal cancer with MR imaging after chemoradiation therapy. Radiographics.

[9] Akiyoshi T, Kobunai T, Watanabe T (2012). Predicting the response to preoperative radiation or chemoradiation by a microarray analysis of the gene expression profiles in rectal cancer. Surg Today.

[10] Grade M, Wolff HA, Gaedcke J, Ghadimi BM (2012). The molecular basis of chemoradiosensitivity in rectal cancer: implications for personalized therapies. Langenbecks Arch Surg.

[11] Lambin P, Rios-Velazquez E, Leijenaar R, Carvalho S, Aerts HJWL (2007). Radiomics: extracting more information from medical images using advanced feature analysis. Eur J Cancer.

[12] Aerts HJ, Velazquez ER, Leijenaar RT, Parmar C, Grossmann P, Carvalho S (2014). Decoding tumour phenotype by noninvasive imaging using a quantitative radiomics approach. Nat Commun.

[13] Bundschuh RA, Dinges J, Neumann L, Seyfried M, Zsoter N, Papp L (2014). Textural parameters of tumor heterogeneity in F-FDG PET/CT for therapy response assessment and prognosis in patients with locally advanced rectal cancer. J Nucl Med.

[14] Lovinfosse P, Polus M, Van Daele D, Martinive P, Daenen F, Hatt M (2018). FDG PET/CT radiomics for predicting the outcome of locally advanced rectal cancer. Eur J Nucl Med Mol Imaging.

[15] Hamerla G, Meyer HJ, Hambsch P, Wolf U, Kuhnt T, Hoffmann KT, et al. Radiomics model based on non-contrast ct shows no predictive power for complete pathological response in locally advanced rectal cancer. Cancers (Basel) 2019;11(11). 10.3390/cancers11111680 PMC6895820 31671766

[16] Zhou X, Yi Y, Liu Z, Cao W, Lai B, Sun K (2019). Radiomics-based pretherapeutic prediction of non-response to neoadjuvant therapy in locally advanced rectal cancer. Ann Surg Oncol.

[17] De Cecco CN, Ganeshan B, Ciolina M, Rengo M, Meinel FG, Musio D (2015). Texture analysis as imaging biomarker of tumoral response to neoadjuvant chemoradiotherapy in rectal cancer patients studied with 3-T magnetic resonance. Investig Radiol.

[18] Boldrini L, Cusumano D, Chiloiro G, Casa C, Masciocchi C, Lenkowicz J (2019). Delta radiomics for rectal cancer response prediction with hybrid 0.35 T magnetic resonance-guided radiotherapy (MRgRT): a hypothesis-generating study for an innovative personalized medicine approach. Radiol Med.

[19] Dinapoli N, Barbaro B, Gatta R, Chiloiro G, Casa C, Masciocchi C (2018). Magnetic resonance, vendor-independent, intensity histogram analysis predicting pathologic complete response after radiochemotherapy of rectal cancer. Int J Radiat Oncol Biol Phys.

[20] Cusumano D, Dinapoli N, Boldrini L, Chiloiro G, Gatta R, Masciocchi C (2018). Fractal-based radiomic approach to predict complete pathological response after chemo-radiotherapy in rectal cancer. Radiol Med.

[21] Li Y, Liu W, Pei Q, Zhao L, Gungor C, Zhu H (2019). Predicting pathological complete response by comparing MRI-based radiomics pre- and postneoadjuvant radiotherapy for locally advanced rectal cancer. Cancer Med.

[22] Yi X, Pei Q, Zhang Y, Zhu H, Wang Z, Chen C (2019). MRI-based radiomics predicts tumor response to neoadjuvant chemoradiotherapy in locally advanced rectal cancer. Front Oncol.

[23] Tang X, Jiang W, Li H, Xie F, Dong A, Liu L (2020). Predicting poor response to neoadjuvant chemoradiotherapy for locally advanced rectal cancer: model constructed using pre-treatment MRI features of structured report template. Radiother Oncol.

[24] Delli Pizzi A, Chiarelli AM, Chiacchiaretta P, d’Annibale M, Croce P, Rosa C (2021). MRI-based clinical-radiomics model predicts tumor response before treatment in locally advanced rectal cancer. Sci Rep.

[25] Boldrini L, Lenkowicz J, Orlandini LC, Dinapoli N, Valentini V (2020). PH-0716: Radiomics pCR predictive model in rectal cancer: an intercontinental validation on real world data. Radiother Oncol.

[26] Edge SB, Compton CC. The American Joint Committee on Cancer: the 7th edition of the AJCC cancer staging manual and the future of TNM. Ann Surg Oncol 2010;17(6):1471–1474. 10.1245/s10434-010-0985-4 20180029

[27] Mandard AM, Dalibard F, Mandard JC, Jacques MMA, Henry-Amar M, Petiot JF, et al. Pathologic assessment of tumor regression after preoperative chemoradiotherapy of esophageal carcinoma. Clinicopathologic correlations. Cancer 1994. 10.1002/1097-0142(19940601)73:11<2680::aid-cncr2820731105>3.0.co;2-c 8194005

[28] Hodapp N (2012). The ICRU Report 83: prescribing, recording and reporting photon-beam intensity-modulated radiation therapy (IMRT)]. Strahlenther Onkol.

[29] Team CR. R: A Language and Environment for Statistical Computing. Computing 2015, Vienna, Austria: R Foundation for Statistical Computing. https://www.r-project.org.

[30] Glimelius B, Beets-Tan R, Blomqvist L, Brown G, Nagtegaal I, Påhlman L (2011). Mesorectal fascia instead of circumferential resection margin in preoperative staging of rectal cancer. J Clin Oncol.

[31] Schmoll HJ, Van Cutsem E, Stein A, Valentini V, Glimelius B, Haustermans K (2012). ESMO Consensus Guidelines for management of patients with colon and rectal cance. A personalized approach to clinical decision making. Ann Oncol.

[32] Tofts PS. Concepts: measurement and MR. Quantitative MRI of the brain: measuring Changes Caused by Disease; 2003.

[33] Lee J, Steinmann A, Ding Y, Lee H, Court LE (2021). Radiomics feature robustness as measured using an MRI phantom. Sci Rep.

[34] Huang YQ, Liang CH, He L, Tian J, Liang CS, Chen X (2016). Development and validation of a radiomics nomogram for preoperative prediction of lymph node metastasis in colorectal cancer. J Clin Oncol.

[35] Rosa C, Caravatta L, Pizzi AD, Di Tommaso M, Cianci R, Gasparini L (2019). Reproducibility of rectal tumor volume delineation using diffusion-weighted MRI: agreement on volumes between observers. Cancer/Radiothérapie.

[36] Delli Pizzi A, Caposiena D, Mastrodicasa D, Trebeschi S, Lambregts D, Rosa C (2019). Tumor detectability and conspicuity comparison of standard b1000 and ultrahigh b2000 diffusion-weighted imaging in rectal cancer. Abdom Radiol.

